# Aflatoxin B1 Impairs TACE Efficacy Through Downregulated Carbonic Anhydrase 2: A Bioinformatics Analysis

**DOI:** 10.1155/ijog/8343866

**Published:** 2026-04-15

**Authors:** Pengsheng Zhang, Yuyun Tong, Xiran Feng, Qi Lan, Jiaping Wang

**Affiliations:** ^1^ Department of Radiology, The Second Affiliated Hospital of Kunming Medical University, Kunming, Yunnan, 650101, China, kmmc.cn

**Keywords:** aflatoxin B1, carbonic anhydrase 2, hepatocellular carcinoma, TACE

## Abstract

Hepatocellular carcinoma (HCC) is one of the leading causes of cancer‐related mortality worldwide, and transcatheter arterial chemoembolization (TACE) remains the core locoregional therapeutic modality for patients with intermediate to advanced disease. However, a considerable proportion of patients exhibit nonresponse to this treatment, which highlights an urgent need to identify reliable predictive biomarkers and elucidate the underlying mechanisms. Environmental carcinogens such as aflatoxin B1 (AFB1) are closely associated with the initiation and progression of liver cancer, yet their impact on the therapeutic efficacy of TACE has not been systematically elucidated. In this study, we integrated transcriptomic profiling, single‐cell RNA sequencing (scRNA‐seq), immune infiltration analysis, and molecular docking techniques to investigate the molecular mechanisms underlying AFB1‐associated TACE nonresponse in HCC. We performed differential expression analysis using public cohorts to screen for genes associated with TACE response status (278 differentially expressed genes [DEGs] identified in the GSE104580 dataset) and tumorigenesis and progression (1365 DEGs identified in the TCGA‐LIHC database), as well as AFB1 exposure. Intersection analysis with the predicted targets of AFB1 was subsequently conducted to identify key regulatory molecules. Functional enrichment analysis was applied to clarify the potential biological pathways involved in TACE nonresponse, and molecular docking was used to evaluate the binding potential between AFB1 and the core target. Our findings revealed that carbonic anhydrase 2 (CA2) could bind stably to AFB1 (Vina score = −7.8 kcal/mol) and possessed diagnostic potential for distinguishing tumor samples from normal ones (AUC = 0.819). Meanwhile, CA2 was identified as the core molecule linking AFB1 exposure to TACE nonresponse, with its expression consistently downregulated in HCC tumor tissues, TACE nonresponders (log_2_FC = −1.063, P.adj = 0.0001), and AFB1‐treated liver cells (log_2_FC = −0.978, P.adj = 0.005). Immune infiltration analysis demonstrated that CA2 expression was significantly correlated with multiple immune cell subsets, suggesting its role in shaping the tumor immune microenvironment. Furthermore, scRNA‐seq and pseudotime trajectory analyses revealed heterogeneous and dynamically downregulated CA2 expression across hepatocyte subpopulations, indicating a potential “gene‐switch” pattern of this gene during the state transition of tumor cells. Collectively, our results suggest that AFB1 may contribute to the development of TACE nonresponse by downregulating CA2, which in turn mediates the regulation of tumor heterogeneity and immune remodeling. This study provides a novel candidate biomarker and a mechanistic framework for optimizing individualized treatment strategies for patients with AFB1‐related HCC.

## 1. Introduction

Hepatocellular carcinoma (HCC) is a leading cause of cancer‐related deaths worldwide [1, 2]. It not only has a continuously increasing incidence but also high mortality and a strong tendency for recurrence, posing a major challenge to human health and public health [3–5]. Early symptoms of HCC are insidious, resulting in most patients being diagnosed at an advanced or late stage, which greatly limits the opportunities for curative treatment. In addition, multiple factors contribute to the occurrence and development of this disease, such as chronic liver diseases (cirrhosis and viral hepatitis), environmental exposures, and diet‐related risks, which complicate its pathological mechanism [6–9]. Although there has been recent progress in early screening and treatment [10], the prognosis for patients with HCC remains poor [11, 12]. Therefore, the identification of reliable prognostic biomarkers for patients with HCC is of crucial clinical significance [13].

Currently, the treatment strategies for HCC are increasingly diversified. For patients diagnosed with early‐stage HCC, radical surgery is the first‐line treatment option [14]. However, for patients with intermediate to advanced HCC, transcatheter arterial chemoembolization (TACE) remains the recognized standard of care for locoregional treatment [15, 16]. By occluding the tumor‐feeding artery and locally releasing chemotherapeutic drugs, TACE provides certain benefits in improving the long‐term survival of patients [17, 18]. Nevertheless, due to the heterogeneity of intermediate‐stage HCC, the response to TACE varies among different patients, leading to the introduction of the concept of TACE refractoriness, which means a nonresponse to TACE treatment [19, 20]. In recent years, research on predicting the appropriate population for TACE treatment has increased. This aims to improve TACE effectiveness, reduce treatment failure caused by inappropriate patient selection, and lower the medical burden of unnecessary invasive procedures. Kazuo Asano et al. evaluated the efficacy of TACE in treating HCC by studying tumor location and tumor burden [21]; Valerie Fako et al. predicted the prognosis of patients undergoing TACE by analyzing tumor gene expression patterns [22]. Moreover, with the development of radiomics technology, an increasing number of imaging techniques and deep learning models have been used to predict the efficacy of TACE [23, 24].

Metabolism‐associated enzymes, as core executors of cellular metabolic networks, exert biological functions that extend far beyond basic energy and material transformation [25]. Accumulating evidence has demonstrated that metabolism‐associated molecules can drive tumor progression and modulate clinical prognosis by regulating canonical oncogenic signaling pathways [26]. Carbonic anhydrases (CAs) constitute a family of enzymes that catalyze the reversible hydration of carbon dioxide. Multiple CA isoforms exert a profound influence on tumor biological behaviors by regulating intracellular and extracellular pH homeostasis [27]. Previous studies have indicated that CA2 is significantly upregulated in HCC tumor tissues, and serum CA2 levels are markedly higher in patients with postoperative recurrence than in those without recurrence. The underlying mechanism is that CA2 overexpression can aberrantly activate the epithelial–mesenchymal transition signaling pathway, thereby significantly enhancing the migratory and invasive capacities of HCC cells. These findings suggest that CA2 holds promise as a novel potential biomarker for the diagnosis and prognostic evaluation of HCC [28]. However, to date, no study has directly elucidated the correlation between CA2 expression levels and the therapeutic efficacy of TACE in the treatment of HCC.

Environmental and foodborne carcinogens play a pivotal role in the development and progression of HCC. Among these, exposure to aflatoxin B1 (AFB1) is particularly prevalent in specific regions, including Sub‐Saharan Africa, Southeast Asia, and China [29]. A large number of previous studies have confirmed that AFB1 can promote the occurrence of HCC through multiple pathways, such as inhibiting DNA repair and inducing gene mutations [9, 30, 31]. Although numerous reports have confirmed that AFB1 greatly promotes the formation of HCC, whether it affects TACE efficacy and how it exerts such effects have not been systematically elucidated. This research gap limits the development of individualized intervention strategies for patients with AFB1‐related HCC who are at risk of TACE nonresponse. Therefore, exploring the impact of AFB1 on TACE efficacy and identifying related biomarkers have become urgent scientific issues to be addressed.

With the development of bioinformatics, new perspectives for predicting biomarkers of TACE efficacy [32] and new perspectives for clarifying the molecular mechanisms of environmental carcinogens have gradually emerged [33]. However, current related studies mostly focus on single transcriptomic analysis. They have not systematically integrated the regulatory mechanisms of the immune microenvironment or tumor heterogeneity. Moreover, these studies have not established the association between AFB1 exposure and TACE nonresponse. Such limitations in current research restrict the interpretation of the mechanisms underlying the differences in TACE efficacy among patients with AFB1‐related HCC. To fill these research gaps, this study integrated transcriptomics, single‐cell RNA sequencing (scRNA‐seq), and molecular docking techniques to investigate the expression patterns and biological functions of key molecular targets through which AFB1 induces TACE nonresponse, evaluate their clinical value as novel diagnostic biomarkers, analyze their roles in immune regulation, and clarify their effects on HCC cell subsets.

## 2. Methods

### 2.1. Data Sources

The TCGA‐LIHC cohort dataset was downloaded from The Cancer Genome Atlas (TCGA, https://portal.gdc.cancer.gov/) portal, which includes 50 normal samples and 424 HCC samples. scRNA‐seq, microarray, and high‐throughput sequencing datasets were acquired from the Gene Expression Omnibus (GEO) database (https://www.ncbi.nlm.nih.gov/gds). Specifically, five tumor region samples (GSM7774399–GSM7774403) were obtained from the scRNA‐seq dataset GSE242889. Data of 81 TACE‐responsive patients and 66 TACE nonresponsive patients were retrieved from the microarray dataset GSE104580, with annotation completed using the GPL570 platform file. Additionally, four aflatoxin‐treated hepatocyte samples (GSM8404956, GSM8404957, GSM8405340, and GSM8405341) and 40 control cell samples treated with 0.1% dimethyl sulfoxide (DMSO) (GSM8404808–GSM8405573) were downloaded from the GSE272576 dataset, with both groups treated for 10 days. Immunohistochemistry (IHC) results were sourced from the Human Protein Atlas (HPA) database (https://www.proteinatlas.org/), an internationally recognized free proteomics database, including IHC results of five patients with specific Patient IDs: 162, 516, 1846, 2652, and 2399 [34]. Gene expression level data associated with different disease stages were acquired from the UALCAN database (https://ualcan.path.uab.edu/) by searching with the keyword “CA2” [35].

### 2.2. Acquisition of Chemical Composition and Targets of AFB1

AFB1 was characterized using multiple databases. Its standardized 2D structural descriptor (SMILES: COC1=C2C3=C(C(=O)CC3)C(=O)OC2=C4[C@@H]5C=CO[C@@H]5OC4=C1) was extracted from the PubChem database. Potential targets of AFB1 were predicted using three databases, including the ChEMBL database (https://www.ebi.ac.uk/chembl/) for ligand–receptor interaction analysis, Swiss Target Prediction (http://swisstargetprediction.ch/) for chemogenomics‐based prediction, and Pharm Mapper (https://lilab-ecust.cn/pharmmapper/index.html) for 3D pharmacophore matching; all predicted targets were derived from the human proteome.

### 2.3. Differential Analysis

Count data from the TCGA‐LIHC and GSE272576 datasets were normalized and subjected to differential expression analysis using the DESeq2 package (Version 1.46.0). For the GSE104580 dataset (comparing TACE response and TACE nonresponse patients), transcriptomic data were analyzed using the limma package (Version 3.62.2). The threshold for differentially expressed genes (DEGs) was set as FDR‐adjusted *p* < 0.05 and |log_2_FC| > 1. Results were visualized using ggplot2.

### 2.4. Identification of AFB1‐Related Disease Targets

The core targets were identified through intersection analysis of three sets of genes: differential genes from the GSE104580 dataset, differential genes from the TCGA‐LIHC dataset, and genes predicted as targets of AFB1. These results were visualized using a Venn diagram.

### 2.5. Functional Enrichment Analysis

Based on the Gene Ontology (GO) [36] and Kyoto Encyclopedia of Genes and Genomes (KEGG) databases [37], DEGs in each dataset were analyzed using the clusterProfiler package (Version 4.4.4) (P.adj <  0.05). The Hallmark gene set was downloaded from the MSigDB Collections database (https://www.gsea-msigdb.org/gsea/msigdb/collections.jsp) for gene set enrichment analysis (GSEA) to explore pathways associated with TACE nonresponse. All results were visualized using the ggplot2 package (Version 3.4.4).

### 2.6. Molecular Docking Analysis

To verify the interaction between AFB1 and the identified core genes, molecular docking was performed using the CB‐DOCK2 platform (https://cadd.labshare.cn/cb-dock2/index.php) [38]. First, ligand structures in SDF format were retrieved from PubChem (https://pubchem.ncbi.nlm.nih.gov/), and 3D protein models of the core targets were obtained from the Protein Data Bank (PDB ID: 1A42). Subsequently, a curvature‐based cavity detection method was used to perform blind docking experiments, which identified potential binding regions on the protein surface, followed by ligand–receptor docking without prior binding site information, and finally, visualization analysis [39].

### 2.7. Expression Profile Analysis and Diagnostic Efficacy Evaluation of CA2

The TCGA‐LIHC dataset was used to analyze the expression levels of CA2 in normal and tumor tissues, with visualization performed using the ggplot2 package (Version 3.4.4) in R. Changes in CA2 expression levels in different disease stages were obtained from the UALCAN database. IHC image data were sourced from the HPA database, where staining intensity reflects protein expression abundance. A receiver operating characteristic (ROC) curve was constructed based on the TCGA dataset, and the area under the curve (AUC) was calculated to evaluate the diagnostic efficacy of CA2. A higher AUC value indicates a stronger ability of CA2 to distinguish between normal and tumor tissues. The specific criteria are as follows: AUC < 0.5 indicates no diagnostic value; AUC > 0.7 is generally considered to have good diagnostic efficacy; AUC > 0.9 indicates excellent diagnostic accuracy. The ROC curve was plotted and the AUC value was calculated using the pROC package (Version 1.18.0) in R to evaluate the feasibility of CA2 as a potential diagnostic biomarker for HCC (and a predictor of TACE nonresponse).

### 2.8. Immune Infiltration Analysis

To explore the role of CA2 in the tumor microenvironment, immune infiltration analysis was performed using TCGA‐LIHC transcriptomic data. The ssGSEA immune infiltration algorithm was used to analyze the association between each sample and 24 types of immune cells [40]. The CIBERSORT algorithm was used to analyze human immune cell subsets and expression matrices, where the gene expression signature sets of 22 immune cell subtypes were obtained from the CIBERSORTx website (https://cibersortx.stanford.edu/) [41].

### 2.9. scRNA‐seq Data Processing, Cell Type Identification, and Pseudotime Analysis

Tissue samples from 5 HCC patients were analyzed in this study. Each sample was converted into a Seurat object using the CreateSeuratObject function of the Seurat package (Version 5.3.0), and quality control was performed for each cell based on the number of genes (400–4000) and mitochondrial gene percentage (< 20%). Subsequently, the data were normalized, and 2000 highly variable genes were screened for further analysis. Principal component analysis (PCA) was used for dimensionality reduction, and the first 20 principal components were selected for subsequent analysis. Clustering was performed using “FindNeighbors” and “FindClusters,” and doublets were removed using DoubletFinder (Version 2.0.6), followed by batch effect removal using the CCA integration method. For visualization and exploratory analysis of the integrated single‐cell data, the Uniform Manifold Approximation and Projection (UMAP) algorithm was used for nonlinear dimensionality reduction. DEGs in each cluster were identified using “FindAllMarkers” with the thresholds of *p* < 0.05 and log_2_FC > 1. Only positive marker genes that were significantly upregulated in the target cluster relative to other clusters were retained (only.pos = TRUE), with a minimum expression percentage of 20% in either the target or control clusters (min.pct = 0.20). Manual annotation of each cluster was performed by matching the top‐ranked cluster‐specific marker genes with classic cell‐type marker genes reported in published literature. HCC cell subsets were then extracted for downstream analysis to explore the expression pattern of CA2 in different cell subsets. To map the differentiation or transformation process among different subtypes of HCC cells, pseudotime trajectory analysis was performed using Monocle software (Version 2.34.0). When constructing the trajectory, highly variable genes were first screened from hepatocytes using the “dispersionTable” function of Monocle software, followed by dimensionality reduction using the DDRTree algorithm. Finally, the trajectory results were visualized.

**FIGURE 1:**
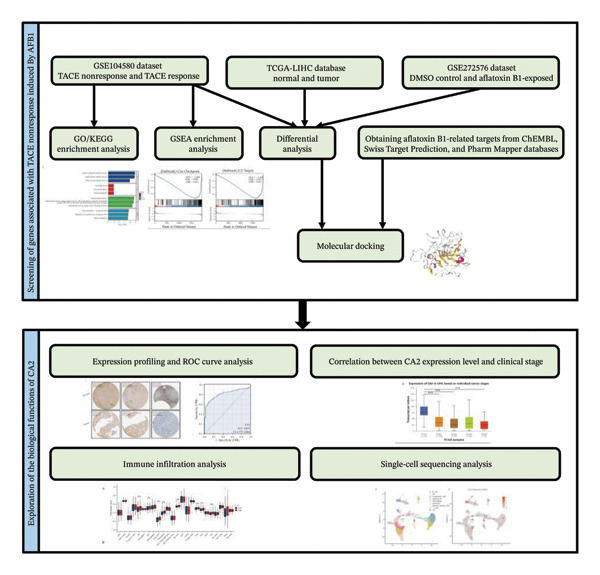
Study workflow.

The technical workflow of this study is shown in Figure 1.

## 3. Results

### 3.1. Identification of Target Genes Related to TACE Nonresponse and Exploration of Their Mechanisms

To clarify the differences in TACE efficacy among patients, we analyzed the GSE104580 dataset, which compares TACE response and nonresponse patients. Using the limma package, we screened a total of 278 DEGs with thresholds of |log_2_FC| > 1 and adjusted *p*‐value (P.adj) < 0.05. Among these, 118 DEGs were downregulated, and 160 were upregulated (Figure 2(a)). Importantly, the expression level of CA2 was significantly upregulated in the TACE responder group (log_2_FC = 1.063, P.adj = 0.0001; Figure 2(a)). To visually present the expression patterns of these DEGs, a heatmap was plotted after Z‐score normalization (Figure 2(b)). To analyze the potential biological functions of the 278 DEGs, GO function and KEGG pathway enrichment analyses were performed with the threshold of P.adj < 0.05. GO enrichment analysis showed that DEGs were mainly enriched in terms such as olefinic compound metabolic process, microsomal cavity, and monooxygenase activity; KEGG enrichment analysis showed that DEGs mainly regulated pathways including drug metabolism–cytochrome P450, cytochrome P450‐mediated xenobiotic metabolism, and retinol metabolism (Figure 2(c)). These pathways may be involved in the development of TACE nonresponse. To further verify the functional characteristics of DEGs from the perspective of “overall gene set trend,” GSEA analysis was performed based on all genes in the GSE104580 dataset, and the three most significant pathways were the G2M checkpoint, E2F targets, and bile acid metabolism (Figure 2(d)).

FIGURE 2Identification of target genes related to TACE nonresponse and exploration of their mechanisms. (a) Volcano plot of differentially expressed genes (DEGs) in the GSE104580 dataset; red indicates upregulated genes, blue indicates downregulated genes, and gray indicates genes with no significant expression change. (b) Heatmap of DEGs in the GSE104580 dataset. (c) Bar plot of Gene Ontology (GO)/Kyoto Encyclopedia of Genes and Genomes (KEGG) enrichment analysis of DEGs in the GSE104580 dataset. (d) Gene set enrichment analysis (GSEA) of genes in the GSE104580 dataset.

**Figure:**
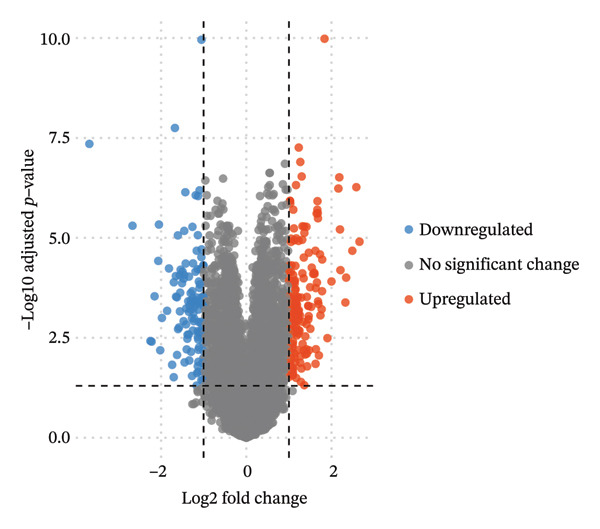
(a)

**Figure:**
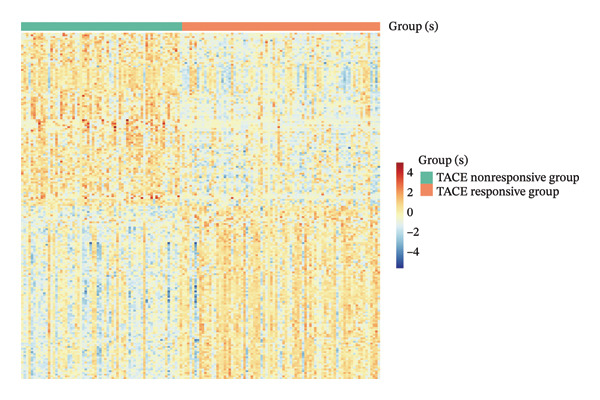
(b)

**Figure:**
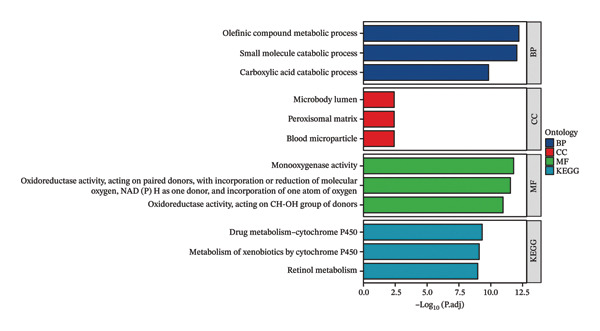
(c)

**Figure:**
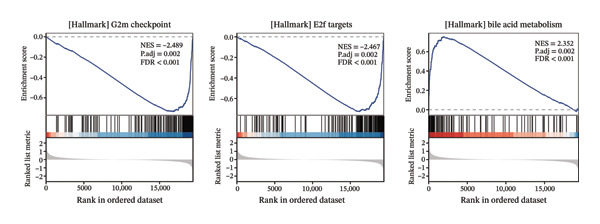
(d)

### 3.2. CA2 as a Target Protein for AFB1 Inducing TACE Nonresponse

The molecular structure of AFB1 was retrieved from the PubChem database (Figure 3(a)). Potential biological targets of AFB1 were systematically predicted using the ChEMBL, Pharm Mapper, and Swiss Target Prediction databases, and intersection analysis with the aforementioned DEG set identified CA2 as a common target (Figure 3(b)). The GSE104580 dataset confirmed that CA2 expression was downregulated in patients with TACE nonresponse (Figure 3(c)). Subsequently, the GSE272576 dataset was used to explore the transcriptomic changes of liver cells exposed to AFB1, and the results showed that CA2 expression was significantly decreased after 10 days of AFB1 treatment (log_2_FC = −0.978, P.adj = 0.005; Figure 3(d)). To further verify the binding relationship between AFB1 and CA2, molecular docking simulation was performed, and the docking results were visualized (Figure 3(e)). The Vina score between them was −7.8 kcal/mol, indicating a strong binding between the small molecule compound (AFB1) and the protein (CA2). The Vina score represents the binding energy between the ligand and CA2, calculated by the AutoDock Vina algorithm. This score is correlated with the experimentally measured binding free energy (ΔG) through a linear regression model, with the specific formula: Δ*G* ≈ VinaScore × *α* + *β*, where *α* (scaling factor) and β (intercept) are derived from the training dataset to align the predicted results with the experimental values. A lower Vina score (more negative value) indicates stronger binding stability between the ligand and the protein, suggesting that AFB1 may stably bind to CA2 to regulate its function and induce TACE nonresponse.

FIGURE 3CA2 as a target protein for aflatoxin B1 inducing TACE nonresponse. (a) Chemical structure of AFB1. (b) Venn diagram of target prediction (for TACE nonresponse) using ChEMBL, PharmMapper, and SwissTargetPrediction. (c) Violin plot of CA2 expression levels in TACE nonresponse and TACE‐effective groups. (d) Heatmap of DEGs in the GSE272576 dataset (AFB1‐treated vs. control) with CA2 gene labeled. (e) Docking interaction between CA2 and aflatoxin B1.

**Figure:**
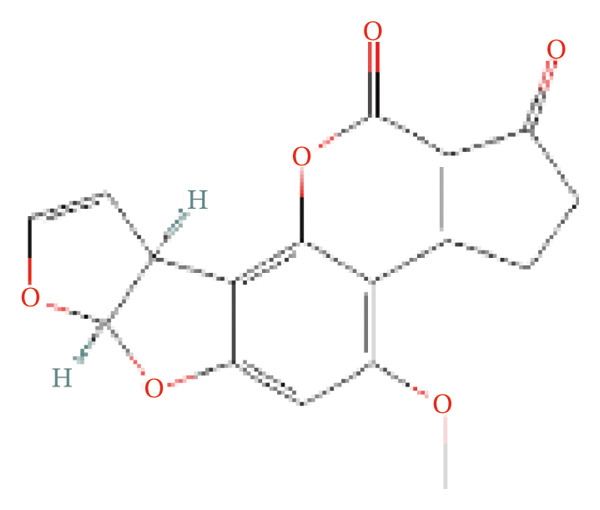
(a)

**Figure:**
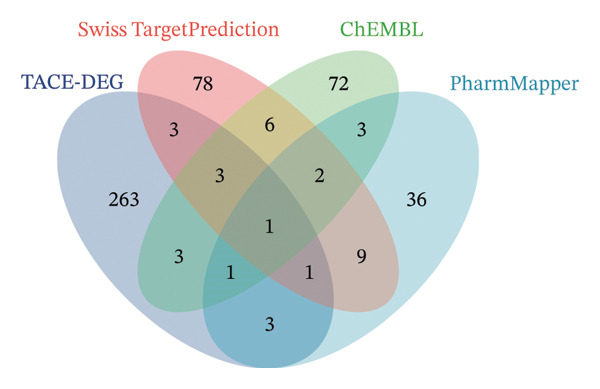
(b)

**Figure:**
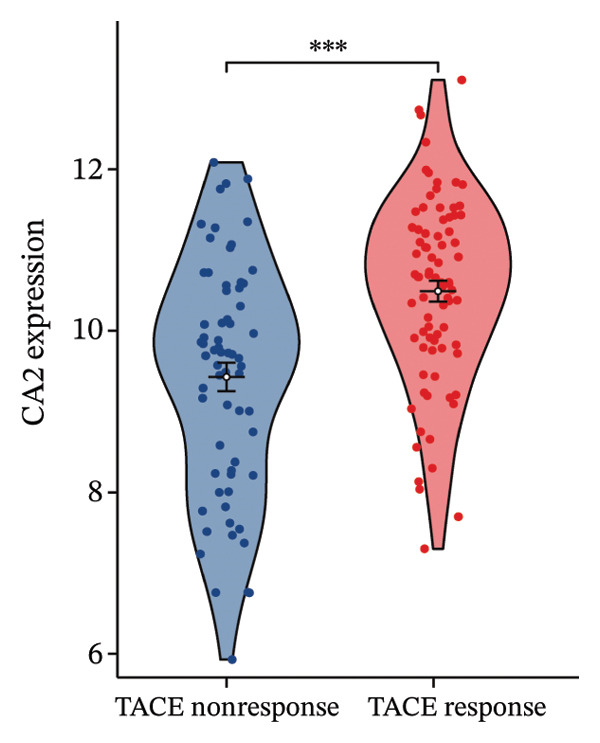
(c)

**Figure:**
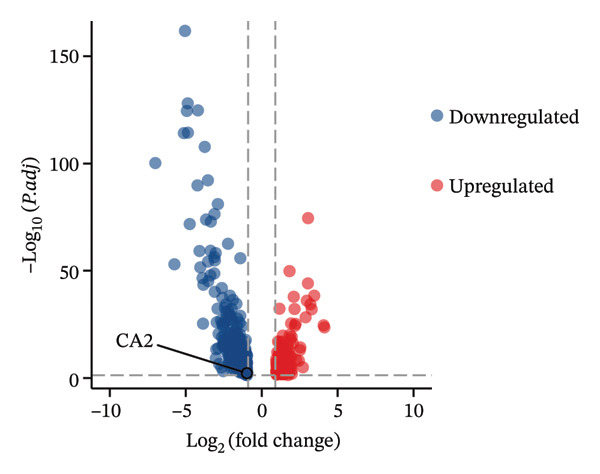
(d)

**Figure:**
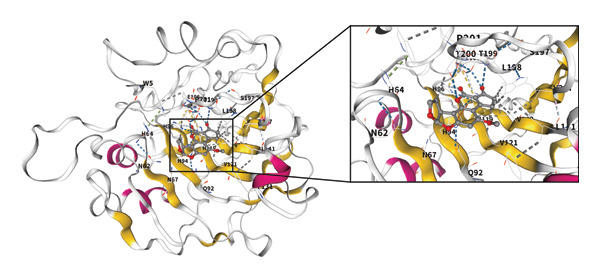
(e)

### 3.3. Expression Level and Diagnostic Value of CA2 in HCC

Differential analysis was performed using the TCGA‐LIHC database with the thresholds of |log_2_FC| > 1 and P.adj < 0.05, resulting in 1365 DEGs, including 1004 downregulated and 361 upregulated genes (Figure 4(a)). Subsequently, the expression level of CA2 was evaluated, and it was found that CA2 expression was significantly downregulated in tumor tissues compared with the control group (*p* < 0.001; Figure 4(b)). IHC results also confirmed this conclusion: The protein expression level of CA2 was lower in tumor samples (Figure 4(c)). To clarify the expression pattern of CA2 during LIHC progression, the transcript levels of CA2 in different clinical stages of TCGA samples were analyzed. With the progression of LIHC stages, CA2 expression showed a downward trend, and CA2 was relatively highly expressed in normal liver tissues. In contrast, CA2 expression was significantly decreased in stage I, II, and IV LIHC samples compared with normal tissues (*p* < 0.001), suggesting that CA2 downregulation may be associated with HCC progression and an increased risk of TACE nonresponse (Figure 4(d)). To further clarify the diagnostic ability of CA2 between HCC tissues and normal tissues, ROC analysis was performed, and CA2 was found to have good diagnostic value (AUC = 0.819; Figure 4(e)).

FIGURE 4Expression level and diagnostic value of CA2 in hepatocellular carcinoma. (a) Heatmap of differential genes in the TCGA‐LIHC database. (b) Violin plot of CA2 expression levels in the normal group and cancer group analyzed by the TCGA‐LIHC database. (c) Representative images of CA2 immunohistochemical staining in normal liver and cancer tissues. (d) Box plot of CA2 expression levels in different stages of HCC. ^∗^, *p* < 0.05; ^∗∗^, *p* < 0.01; ^∗∗∗^, *p* < 0.001; ^∗∗∗∗^, *p* < 0.0001. (e) Receiver operating characteristic (ROC) curve showing the diagnostic efficacy of CA.

**Figure:**
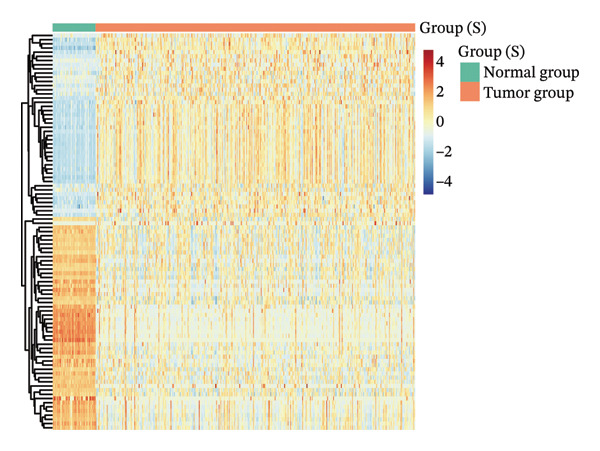
(a)

**Figure:**
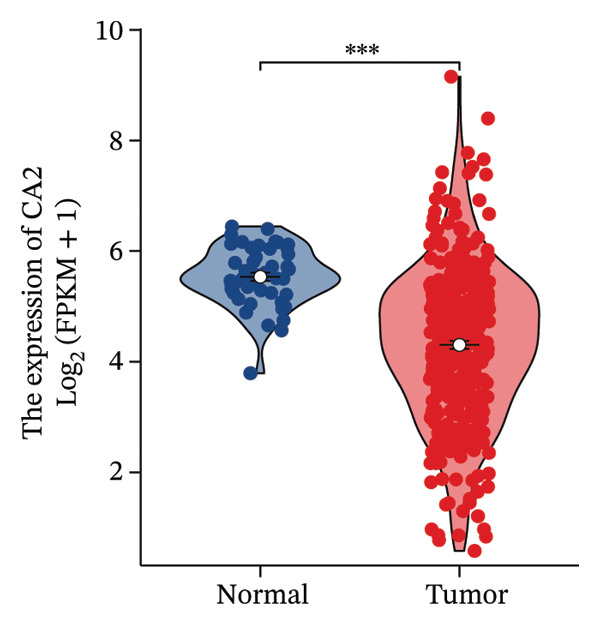
(b)

**Figure:**
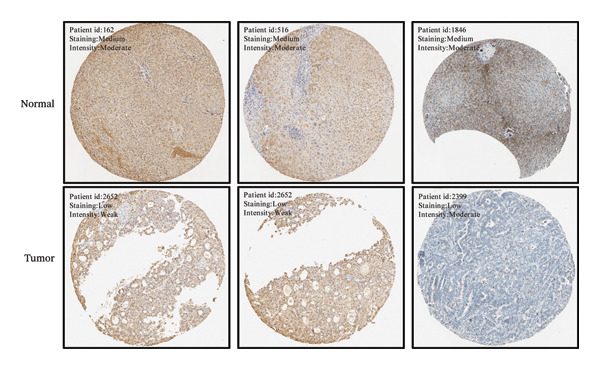
(c)

**Figure:**
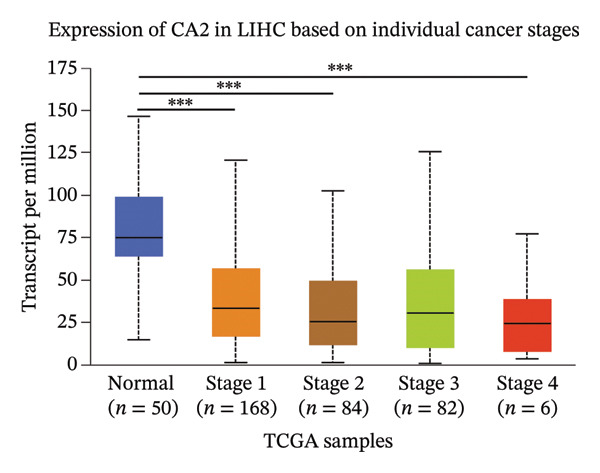
(d)

**Figure:**
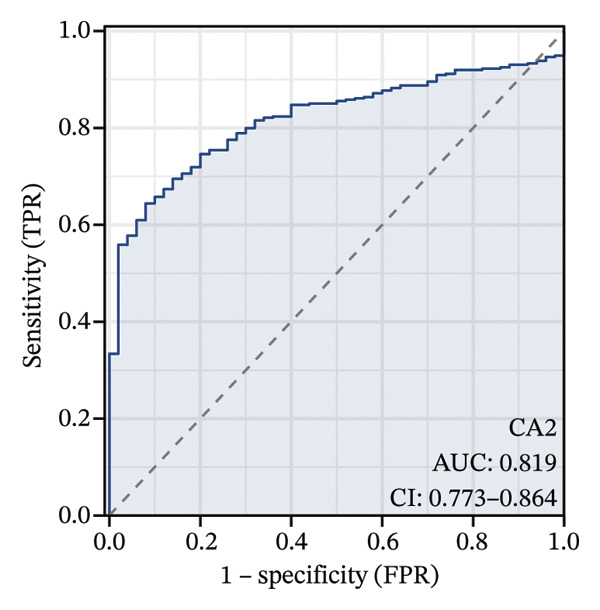
(e)

### 3.4. Relationship Between CA2 Expression Level and Tumor‐Infiltrating Immune Cells

HCC patients in the TCGA‐LIHC dataset were divided into high‐expression and low‐expression groups based on the median CA2 expression level. The ssGSEA algorithm was used to detect the tumor infiltration of 24 types of immune cells. Immune cells showing differences in infiltration between the high‐expression and low‐expression groups included DC, iDC, mast cell, neutrophils, NK cells, Tcm, Tem, and Tgd (Figure 5(a)). Meanwhile, the tumor infiltration of 24 types of immune cells in HCC was analyzed, and their correlation with CA2 expression level was evaluated using Spearman’s correlation coefficients. CA2 was positively correlated with Tgd (*R* = 0.228), neutrophils (*R* = 0.227), NK cells (*R* = 0.209), Tcm (*R* = 0.203), mast cells (*R* = 0.148), Tem (*R* = 0.138), DC (*R* = 0.117), and iDC (*R* = 0.112) (Figure 5(c)). In contrast, NK CD56 bright cells (*R* = −0.137) were negatively correlated with CA2 (Figure 5(c)). Using the same method, the CIBERSORT algorithm was used to re‐evaluate the immune infiltration of 22 immune cell subtypes in HCC patients. Among these 22 immune cell subtypes, immune cells showing differences in infiltration between the high‐expression and low‐expression groups included T cells CD8, T cells CD4 memory resting, T cells follicular helper, T cells gamma delta, and macrophages M2 (Figure 5(b)). Spearman correlation analysis showed that CA2 was positively correlated with macrophages M2 (*R* = 0.146), T cells CD4 memory resting (*R* = 0.142), and macrophages M1 (*R* = 0.107) (Figure 5(d)), but negatively correlated with T cells CD4 memory activated (*R* = −0.107), T cells gamma delta (*R* = −0.128), T cells CD8 (*R* = −0.129), T cells regulatory Tregs (*R* = −0.138), and T cells follicular helper (*R* = −0.238) (Figure 5(d)). These results suggest that CA2 may regulate the tumor immune microenvironment by altering immune cell infiltration, thereby affecting TACE efficacy and inducing TACE nonresponse.

FIGURE 5Relationship between CA2 expression level and tumor‐infiltrating immune cells. (a) Box plot of immune cell infiltration differences between CA2 high‐expression and low‐expression groups evaluated by the ssGSEA algorithm. (b) Box plot of immune cell infiltration differences between CA2 high‐expression and low‐expression groups evaluated by the CIBERSORT algorithm. (c) Lollipop plot of the correlation between CA2 expression level and immune cell infiltration level evaluated by the ssGSEA algorithm. (d) Lollipop plot of the correlation between CA2 expression level and immune cell infiltration level evaluated by the CIBERSORT algorithm.

**Figure:**
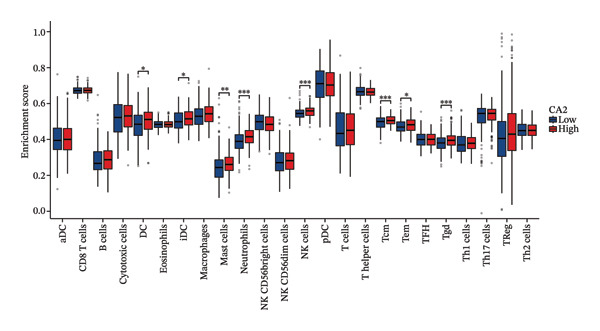
(a)

**Figure:**
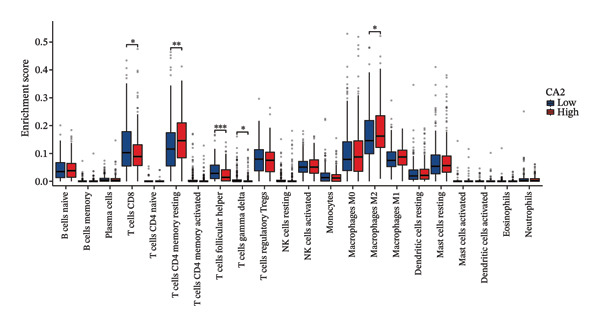
(b)

**Figure:**
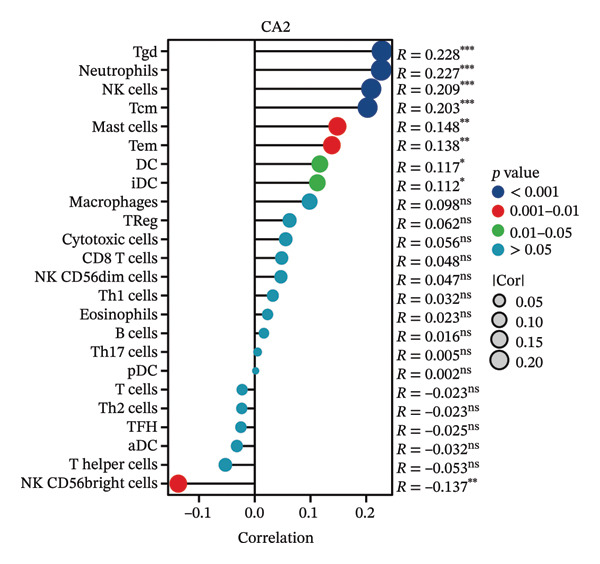
(c)

**Figure:**
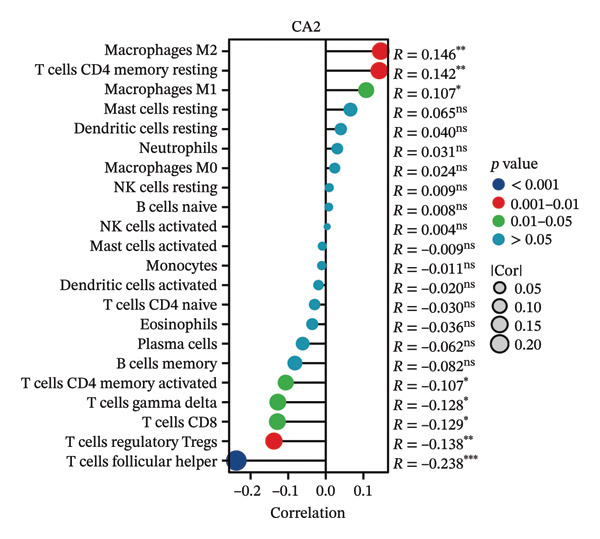
(d)

### 3.5. Characteristic Analysis of CA2 in HCC Based on Single‐Cell Sequencing

In addition, single‐cell transcriptomics technology was used to deeply study the gene expression profiles and interactions of various cell components in HCC tissues. To ensure the reliability of subsequent analysis, strict quality control was first performed on the scRNA‐seq data. Quality control indicators included the number of detected genes per cell (nFeature), total transcript count (nCount), and mitochondrial gene expression ratio, with results presented as a violin plot (Figure 6(a)). Through UMAP dimensionality reduction and clustering analysis, the cells after quality control were divided into 19 cell clusters (Figure 6(b)).

FIGURE 6Characteristic analysis of CA2 in hepatocellular carcinoma based on single‐cell sequencing. (a) Violin plot of quality control indicators (nFeature, nCount, and mitochondrial gene percentage) of 5 HCC samples in the single‐cell transcriptome sequencing dataset. (b) UMAP visualization of cell clustering results of 5 HCC samples in the single‐cell transcriptome sequencing dataset. (c) Heatmap of the expression of cell cluster marker genes in the single‐cell transcriptome sequencing dataset. (d) Bar plot of the proportion of each cell type in different HCC samples in the single‐cell transcriptome sequencing dataset. (e) UMAP visualization of annotated major cell types in HCC samples. (f) Analysis plot of CA2 expression levels in different cell types in the single‐cell transcriptome sequencing dataset.

**Figure:**
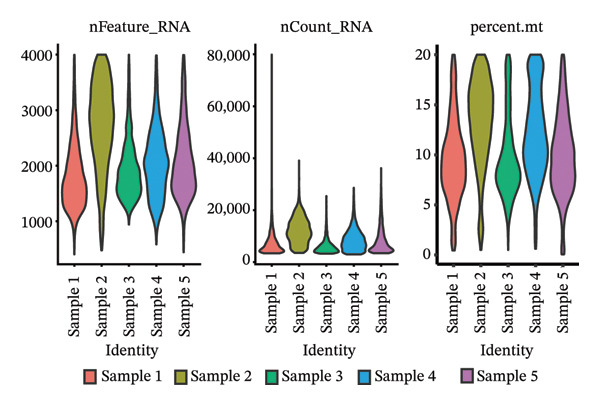
(a)

**Figure:**
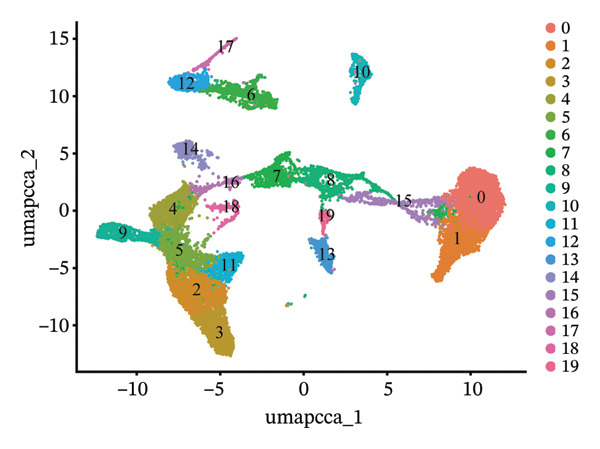
(b)

**Figure:**
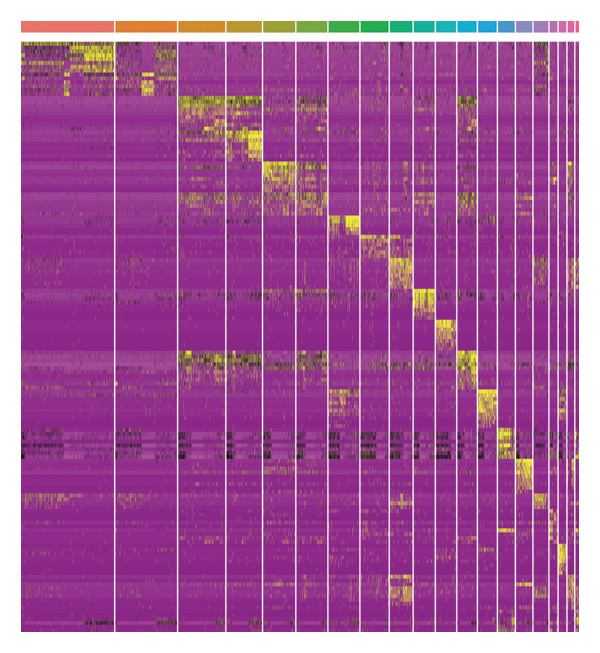
(c)

**Figure:**
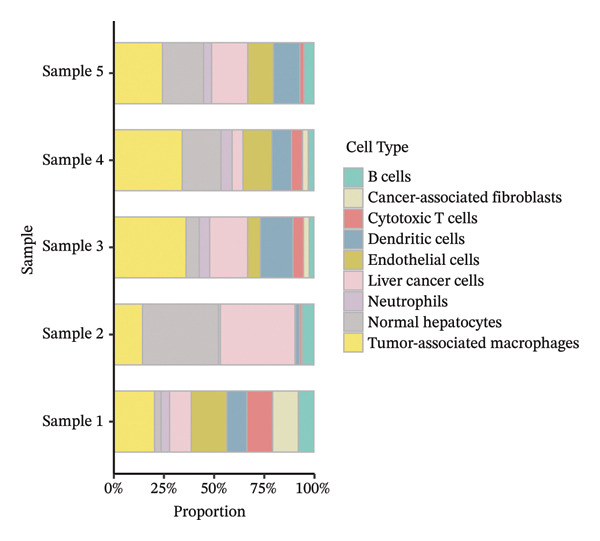
(d)

**Figure:**
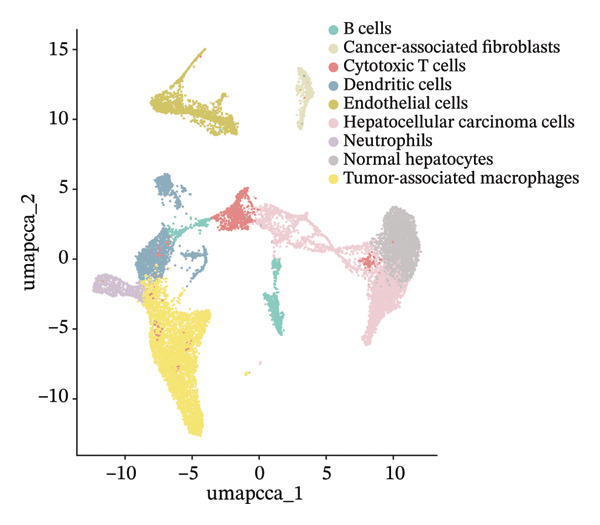
(e)

**Figure:**
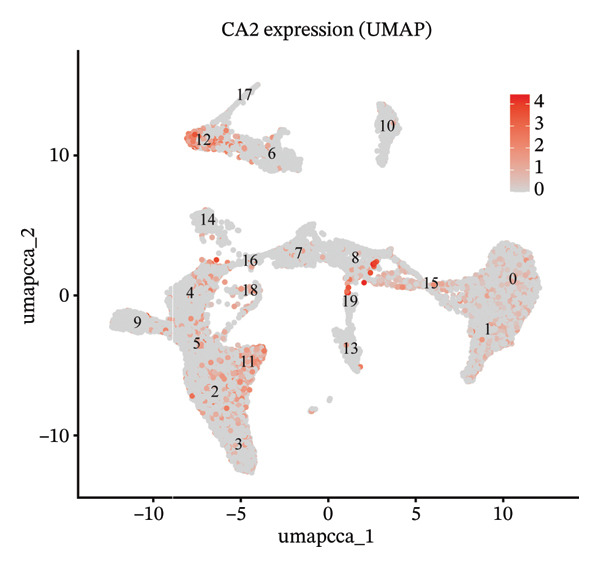
(f)

To clarify the biological identity of each cell cluster, manual annotation of all cell clusters was completed by matching the top‐ranked cluster‐specific marker genes with the classic cell‐type marker genes reported in published literature. Meanwhile, core marker genes for each cluster were screened by integrating differential expression analysis, and the expression patterns of the top 10 marker genes were visualized as a heatmap (Figure 6(c)). Ultimately, nine major cell types were annotated (Figure 6(e)). To clarify the differences in cell type composition among different samples, the number proportion of each of the 9 cell types was calculated and presented as a bar plot (Figure 6(d)). Meanwhile, the expression of CA2 in different clusters was explored, and the results showed that CA2 expression varied among different HCC cell clusters (Figure 6(f)), suggesting that CA2 may play cell‐type‐specific roles in mediating TACE nonresponse.

### 3.6. Pseudotime Analysis of HCC Cell Subsets

Through UMAP analysis, all HCC cells were divided into three subtypes (HCC cell Subtypes 1, 2, and 3), and the differences in marker genes among the three subtypes were presented as a heatmap (Figure 7(c)). GO and KEGG analyses showed that characteristic genes in HCC cell Subtype 1 were mainly enriched in “metabolism”‐related functions, Subtype 2 in “tumor stroma”‐related functions, and Subtype 3 in “immune regulation”‐related functions (Figure 7(a)). These functional differences may be associated with TACE nonresponse. Analysis showed that HCC cells with high CA2 expression were mainly Subtype 2, while those with low CA2 expression were mainly Subtype 1 and Subtype 3 (Figures 7(b), 7(d)). Subsequently, Monocle software (Version 2.34.0) was used to infer the cell state trajectory, thereby exploring the dynamic state and cell transformation process of hepatocytes. The analysis results showed that the HCC cell cluster consisted of three stages. Subtype 2 was mainly in Stage 1, Subtype 1 in Stage 3, and Subtype 3 in Stage 2 (Figure 7(g)). Hepatocyte Subtype 2 was located at the starting position of this trajectory path and gradually transformed into Subtype 1 and Subtype 3, with a branch point appearing (Figures 7(e), 7(f)). CA2 expression was higher in hepatocytes at Stage 1, while lower in hepatocytes at Stage 2 and Stage 3, suggesting the existence of a “Gene Switch” phenomenon in HCC cells (Figure 7(h)). This dynamic change in CA2 expression may be involved in the transformation of HCC cell subtypes and the development of TACE nonresponse.

FIGURE 7Pseudotime analysis of hepatocellular carcinoma cell subsets. (a) Bar plot of GO and KEGG enrichment analysis of marker genes after reclustering of hepatocyte subsets. (b) UMAP visualization of reclustered hepatocyte subsets. (c) Heatmap of the expression of reclustered hepatocyte subset marker genes. (d) Analysis plot of CA2 expression levels in hepatocyte subsets. (e) Dimensionality reduction plot of single‐cell pseudotime analysis based on Monocle 2. Pseudotime values from dark to light indicate the progression of cells along the differentiation trajectory, showing the continuous differentiation process of cells from the initial state to the terminal state. (f) Dimensionality reduction plot of single‐cell pseudotime analysis based on Monocle 2, showing the distribution differences of different subtype cells in the differentiation trajectory. (g) Dimensionality reduction plot of single‐cell pseudotime analysis based on Monocle 2, showing the clustering and transition of cells in different states in the trajectory. (h) Pseudotime dynamic plot of single‐cell gene expression: the horizontal axis represents the relative gene expression level (relative expression), the vertical axis represents pseudotime (pseudotime), and red, green, and blue represent different differentiation states (States 1–3), showing the changes in CA2 expression patterns during cell differentiation.

**Figure:**
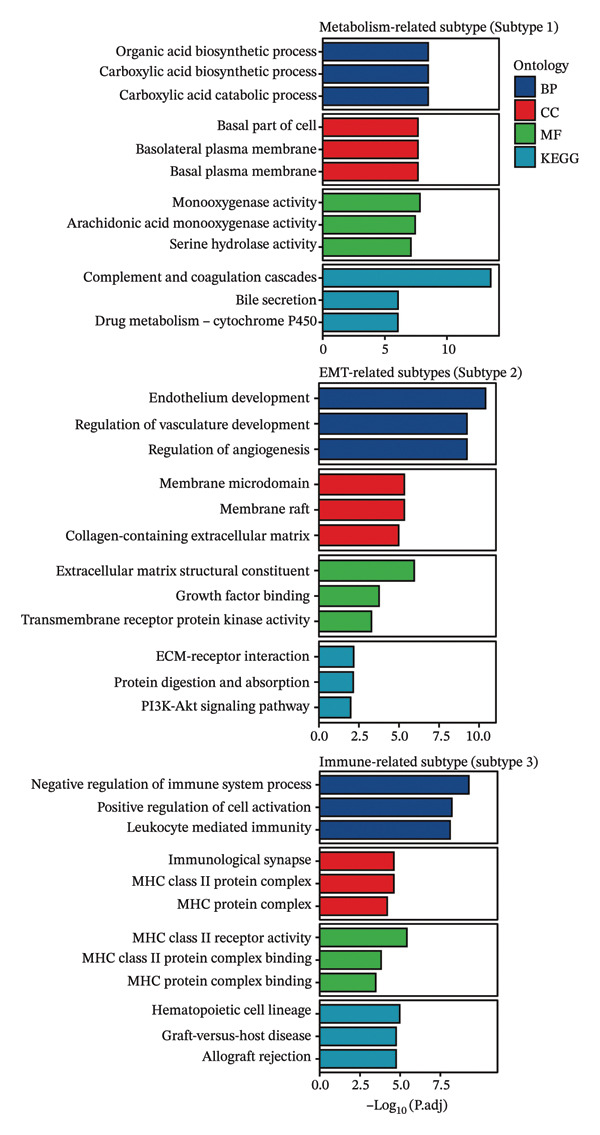
(a)

**Figure:**
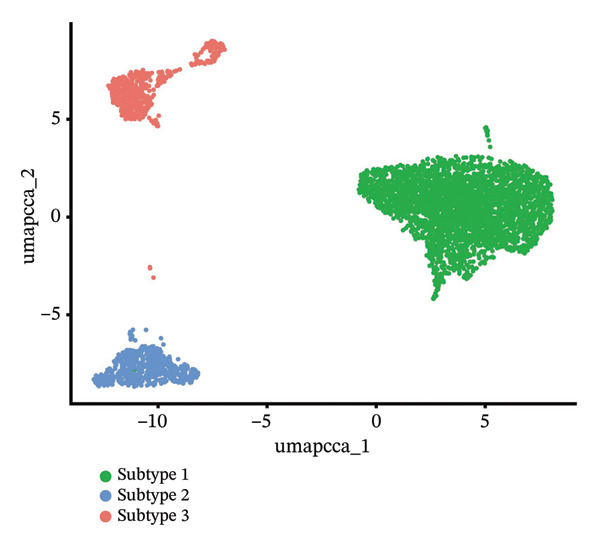
(b)

**Figure:**
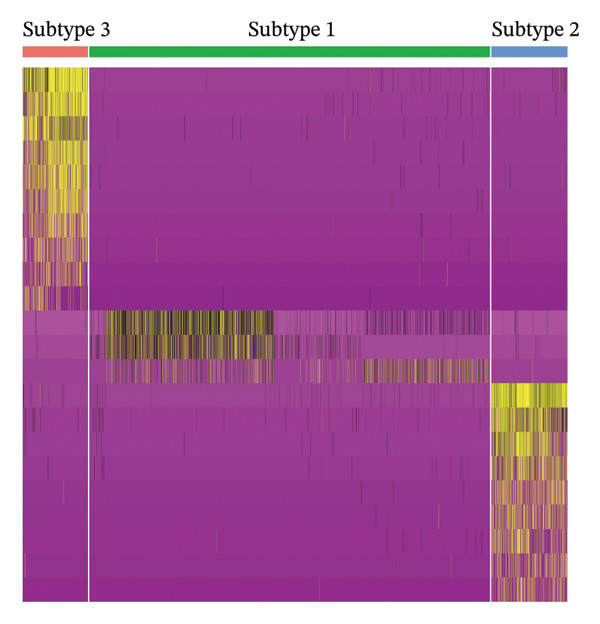
(c)

**Figure:**
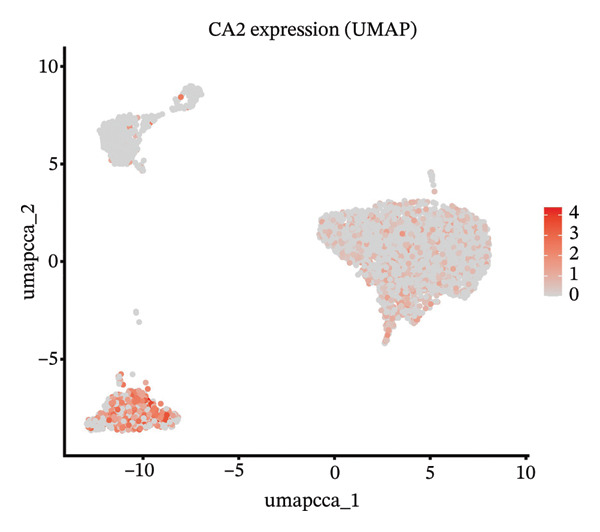
(d)

**Figure:**
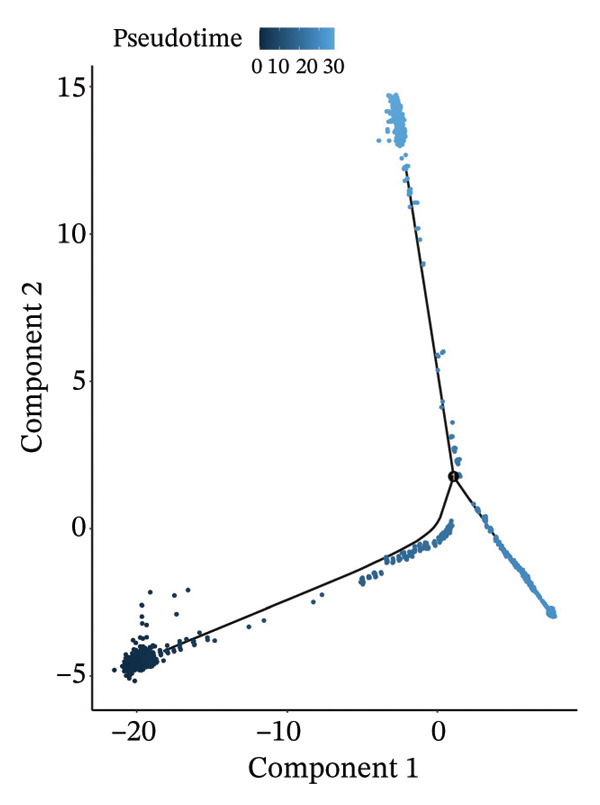
(e)

**Figure:**
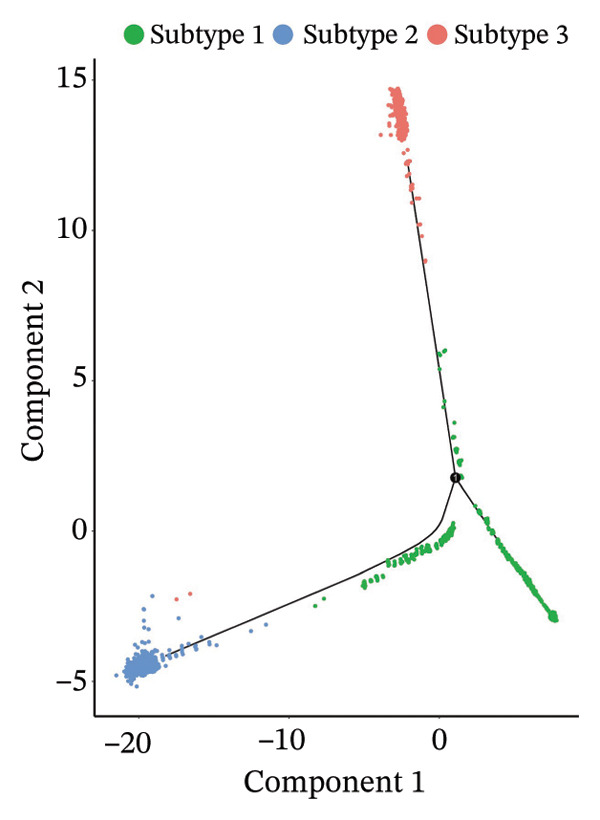
(f)

**Figure:**
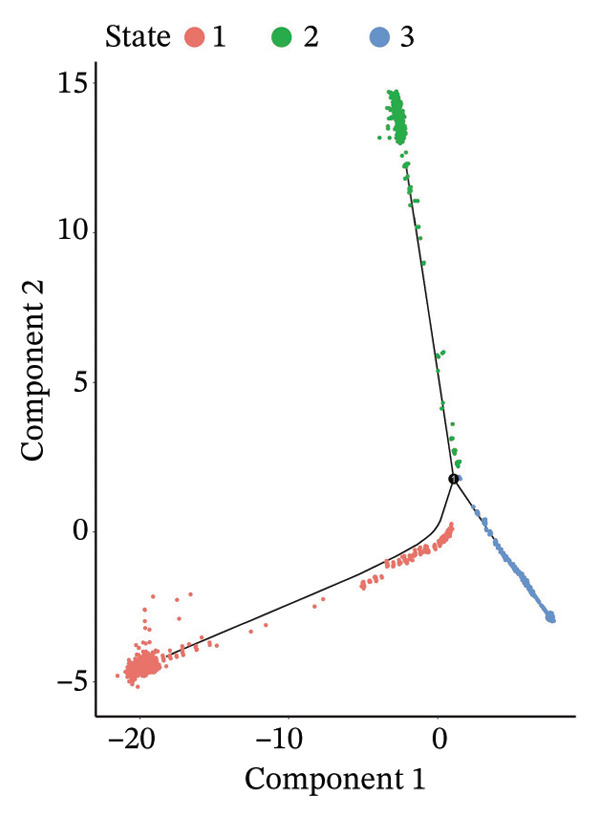
(g)

**Figure:**
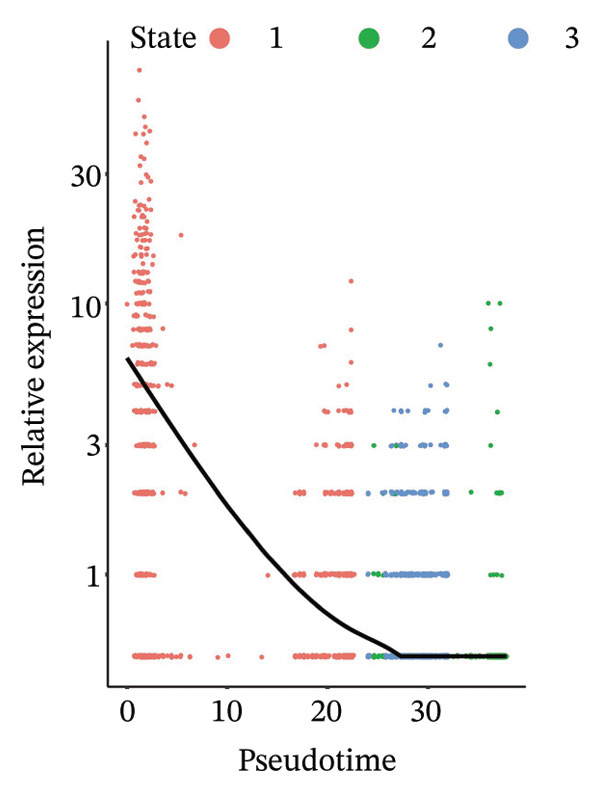
(h)

## 4. Discussion

Currently, regardless of whether TACE is used as a first‐line treatment or as a key component of combined therapy or conversion therapy, at least 30% of HCC patients receive TACE during treatment [42]. However, even with the same TACE regimen for HCC patients, significant heterogeneity may be observed in survival benefits among different individuals, and a considerable proportion of patients develop TACE nonresponse. Most previous studies have focused on constructing a prediction system for TACE efficacy in HCC patients based on imaging features, identifying transcriptomic/proteomic biomarkers, or analyzing clinical characteristics. However, research into the mechanisms of TACE nonresponse at the tumor cell level across different patients remains relatively scarce, and no targeted analysis has been conducted on how AFB1 affects the efficacy of TACE. In this study, by integrating the results of expression verification, functional enrichment, and single‐cell transcriptome analysis, we found that AFB1 affects tumor cell heterogeneity and the immune microenvironment through CA2, thereby leading to TACE nonresponse, providing a new perspective for interpreting AFB1‐induced TACE nonresponse.

Through differential analysis and gene function enrichment analysis of the GSE104580 dataset (comparing TACE response and TACE nonresponse patients), we found that DEGs were mainly enriched in terms such as olefinic compound metabolic process, carboxylic acid catabolic process, and drug metabolism–cytochrome P450 (Figure 1(c)). This result suggests that changes in the endogenous metabolic characteristics of tumor cells may be closely related to the mechanism of TACE nonresponse. Currently, the concept of metabolic reprogramming in cancer cells has long been recognized as an “emerging hallmark” of cancer [43, 44]. The research results of Sean P Martin et al. showed that glucose metabolism disorders are heterogeneous among different HCC patient populations, and PKM2 expression affects glycolytic activity, thereby leading to TACE nonresponse [45]. This explains the differences in TACE efficacy among different individuals to a certain extent.

AFB1, the most toxic form of aflatoxin, has a clear multilevel association with the olefinic compound metabolic process. The AFB1 molecule can be catalyzed by the liver cytochrome P450 enzyme system as a substrate, generating the highly active AFB1‐8,9‐epoxide (AFBO) through oxidation, which is the core effector molecule of AFB1 toxicity [46]. The research results of Xi et al. showed that mutations in the xeroderma pigmentosum complementation group C (XPC) gene disrupt XPC gene expression and promote the hepatocarcinogenic effect of AFB1, and XPC interacts with AFB1 during HCC carcinogenesis, and this interaction affects the prognosis of HCC [47]. Therefore, we explored the relationship between aflatoxin‐related genes and TACE nonresponse‐related genes, and the results showed that CA2, as their common molecule, mediates AFB1‐induced TACE nonresponse (Figure 2(b)). Through differential analysis of the GSE272576 dataset, the results showed that CA2 was significantly downregulated in hepatocytes treated with AFB1 (Figure 2(d)), which was consistent with the downregulation of CA2 in patients with TACE nonresponse (Figure 2(c)). Meanwhile, using the TCGA‐LIHC database, we also verified that CA2 expression was indeed downregulated in HCC patients (Figures 3(a), 3(b), 3(c)), and the expression level of CA2 could be used to diagnose HCC patients to a certain extent (Figure 3(e)).

As a member of the CA family, CA2 is involved in the regulation of intracellular and extracellular pH homeostasis and CO_2_ metabolism. Its downregulation may lead to the aggravation of the acidic microenvironment of tumor cells, thereby enhancing chemoresistance and immunosuppressive effects [48, 49]. Lu et al. further demonstrated that sorafenib treatment downregulates monocarboxylate transporter 4 (MCT4), which in turn triggers intracellular lactate accumulation and a concomitant drop in pH in HCC cells; notably, high CA2 expression can counteract such MCT4‐mediated pH dysregulation, effectively maintaining intracellular pH homeostasis to support cancer cell survival and drive the development of sorafenib resistance. Moreover, targeted inhibition of CA2 with the clinical agent dorzolamide was shown to restore sorafenib sensitivity in drug‐resistant HCC cells, which highlights the promising translational potential of sorafenib combined with CA2 inhibition for the management of refractory HCC [49].

In addition, some reports have shown that local tissue hypoxia and acidification after HCC chemotherapy promote the autophagy and anti‐apoptotic ability of tumor cells [50]. The downregulation of CA2 may regulate the local acid–base balance, promote the metabolic adaptation of tumor cells after TACE, and thus lead to TACE nonresponse. At the same time, this study revealed that CA2 levels are closely related to the infiltration of various immune cells, especially Tgd cells, neutrophils, and NK cells. Previous studies have shown that changes in the tumor‐related immune microenvironment are closely related to HCC progression and chemotherapy response [51], and the expression of certain metabolic enzymes can directly affect the recruitment and function of immune cells [52].

In recent years, with the development of single‐cell sequencing, our understanding of tumor types has deepened [53–55]. The research results of Tan et al. showed that TREM2^+^ tumor‐associated macrophages (TAMs) play an important role in inhibiting CD8^+^ T cells, explaining the recurrence and progression of HCC after TACE from the perspective of the tumor immune microenvironment [56]. In addition, a study on mouse models showed that CTNNB1 mutations are not only associated with the progression of HCC but also with TACE nonresponse; meanwhile, this study further confirmed through single‐cell analysis that tumor heterogeneity is a key factor leading to treatment failure and nonresponse [57]. However, the applicability of this research result obtained based on animal models under human physiological conditions remains to be verified. In our study, the expression heterogeneity of CA2 among different HCC cell subtypes was further clarified. The dynamic expression changes of CA2 reveal its potential role in regulating the function of HCC cells during transformation and development, providing a new perspective for interpreting the mechanism of treatment failure and nonresponse caused by tumor heterogeneity in AFB1‐related HCC.

While these findings enhance our understanding of the mechanisms by which tumor heterogeneity contributes to AFB1‐mediated TACE nonresponse, this study has limitations. First, although multiple public database datasets were used for analysis, in vitro or in vivo verification is lacking, which may lead to the lack of biological reproducibility of our findings and verification of clinical application. Second, batch effects may exist between different datasets, which affects our evaluation of CA2 expression levels and their correlation with immune cell infiltration, thereby influencing the interpretation of results. Finally, data on the correlation between CA2 expression levels and the prognosis of patients with TACE nonresponse are still scarce. Therefore, additional exploratory cohorts incorporating prognostic information are urgently needed for further research. In conclusion, this study provides new insights into the mechanisms underlying aflatoxin‐induced TACE nonresponse and proposes potential strategies to improve treatment outcomes.

## 5. Conclusion

This study evaluated the key regulatory genes through which AFB1 affects TACE efficacy in different HCC patients based on multiple databases and analyzed their roles in the tumor immune microenvironment and heterogeneity. The study found that the target gene CA2 may be involved in AFB1‐mediated TACE nonresponse and revealed the potential cellular‐molecular mechanisms underlying this process. However, it should be noted that this study mainly relied on computational biology methods. Although the biological rationality of the results was verified through multidimensional data analysis, the specific molecular mechanism by which CA2 mediates AFB1‐induced TACE nonresponse has not yet been validated using in vitro cell experiments and in vivo animal models. Additionally, there is a lack of large‐sample clinical cohort data to support the association between CA2 expression and TACE efficacy or prognosis in patients. Therefore, there remains a gap between the current computational prediction results and the biological mechanisms in real clinical scenarios. Future studies should integrate large‐sample clinical follow‐up data, combine in vitro and in vivo experiments to verify the role of CA2, and explore its downstream pathways. This will provide a reliable basis for reversing TACE nonresponse and achieving precise treatment in AFB1‐related HCC patients.

## Author Contributions

Pengsheng Zhang was responsible for writing–original draft, writing–review and editing, software, data curation, conceptualization, investigation, and formal analysis. Yuyun Tong was responsible for writing–review and editing, methodology, investigation, funding acquisition, data curation, and conceptualization. Xiran Feng was responsible for writing–review and editing and software. Qi Lan was responsible for writing–review and editing. Jiaping Wang was responsible for writing–review and editing, methodology, investigation, funding acquisition, data curation, and conceptualization. Pengsheng Zhang, and Yuyun Tong have contributed equally.

## Funding

This work was supported by the Scientific Research Foundation Project of Yunnan Provincial Department of Education (Grant No. 2026Y0364), the Science and Technology Plan Project of Yunnan Provincial Department of Science and Technology (Grant No. 202501AY070001‐249), and the Science and Technology Plan Project of Yunnan Provincial Department of Science and Technology (Grant No. 202401AY070001‐005).

## Ethics Statement

This study utilizes open‐access databases and does not engage in original research involving human participants or animals.

## Conflicts of Interest

The authors declare no conflicts of interest.

## Data Availability

The TCGA‐LIHC cohort dataset was downloaded from The Cancer Genome Atlas (TCGA, https://portal.gdc.cancer.gov/) portal. Single‐cell RNA sequencing, microarray, and high‐throughput sequencing datasets were acquired from the Gene Expression Omnibus (GEO) database (https://www.ncbi.nlm.nih.gov/gds), with their respective accession numbers GSE242889, GSE104580, and GSE272576. Immunohistochemistry (IHC) results were sourced from the Human Protein Atlas (HPA, https://www.proteinatlas.org/), with specific Patient IDs: 162, 516, 1846, 2652, and 2399. Gene expression level data associated with different disease stages were obtained by searching the UALCAN database (https://ualcan.path.uab.edu/) using the keyword “CA2.”
